# Drawing Firmer Conclusions: Autistic Children Show No Evidence of a Local Processing Bias in a Controlled Copying Task

**DOI:** 10.1007/s10803-016-2889-z

**Published:** 2016-08-17

**Authors:** Alastair D. Smith, Lorcan Kenny, Anna Rudnicka, Josie Briscoe, Elizabeth Pellicano

**Affiliations:** 1 School of Psychology, University of Nottingham, University Park, Nottingham, NG7 2RD UK; 2 Centre for Research in Autism and Education (CRAE), UCL Institute of Education, University College London, London, UK; 3 Department of Experimental Psychology, University of Bristol, Bristol, UK; 4 School of Psychology, University of Western Australia, Perth, Australia

**Keywords:** Autism, Drawing, Global, Local, Coherence, Grouping

## Abstract

Drawing tasks are frequently used to test competing theories of visuospatial skills in autism. Yet, methodological differences between studies have led to inconsistent findings. To distinguish between accounts based on local bias or global deficit, we present a simple task that has previously revealed dissociable local/global impairments in neuropsychological patients. Autistic and typical children copied corner elements, arranged in a square configuration. Grouping cues were manipulated to test whether global properties affected the accuracy of reproduction. All children were similarly affected by these manipulations. There was no group difference in the reproduction of local elements, although global accuracy was negatively related to better local processing for autistic children. These data speak against influential theories of visuospatial differences in autism.

## Introduction

Drawing is a common everyday activity that is unique to humans (La Femina et al. 2009). Graphic production is particularly interesting because it requires synthesis of a number of component functions, including visual perception, mental imagery, memory, attention and action (see Smith 2009). Accordingly, drawing is commonly used as a laboratory tool to study a variety of behavioural features, from the development of reasoning abilities in childhood (e.g., Preissler and Bloom 2008) to the decline of motor coordination in old age (e.g., Morgan et al. 1994). It is for these reasons that drawing production has proved to be an important facet of autism research.

Cognitive and perceptual processing has long been known to be unusual in autistic individuals (Frith 1989). Performance on visuospatial tasks appears to illustrate a particular profile of strengths *and* weaknesses, with participants on the autism spectrum sometimes performing at a level superior to that of typical individuals of similar age and ability (for review, see Simmons et al. 2009). These strengths have been demonstrated in tasks such as pattern discrimination (Plaisted et al. 1998), block design (Shah and Frith 1993) and detecting embedded figures (Jolliffe and Baron-Cohen 1997; Pellicano et al. 2006).

It is likely that interest in assessing drawing behaviour in autistic people was initially sparked by observations of artistic savant abilities in some individuals (e.g., Mottron and Belleville 1995; Selfe 1977). Such cases are rare, although group-based studies of drawing behaviour in autism have also reported superior performance relative to typical individuals. For example, Mottron et al. (1999) conducted a copying task, where autistic adults and adolescents were required to reproduce impossible figures (i.e., three-dimensional designs where the global form does not conform to geometric rules, but the local parts do). Both groups copied possible figures with equal speed and accuracy, but autistic individuals copied impossible figures significantly faster than typical individuals. Another study by Sheppard et al. (2007) required autistic children and adolescents to copy meaningful and non-meaningful figures (constructed using the same line components) that were either two-dimensional or three-dimensional. Both autistic and typical groups produced more accurate copies of meaningful figures, compared to non-meaningful, and also of two-dimensional, compared to three-dimensional stimuli. Importantly, however, autistic children were less affected by dimensionality than typical children, and more accurately depicted projection and perspective for three-dimensional figures. This is in line with another study (Sheppard et al. 2009a), which found that autistic participants were less affected by dimensionality than typical participants when copying line-drawn figures, but not real objects.

Explanations for autistic individuals’ greater accuracy in copying tasks have focused on a potential bias towards processing local perceptual features. The weak central coherence (WCC) account (Frith 1989; Frith and Happé 1994; Happé and Frith 2006) is based on the behavioural tendency to focus on details, at the expense of a coherent global gestalt. This propensity is held to occur at the perceptual level, but might also be manifest in more cognitive behaviours, such as extracting gist in language. In contrast, the Enhanced Perceptual Functioning (EPF) account (Mottron and Burack 2001; Mottron et al. 2006; see also Wang et al. 2007) specifically posits that superiorities in information processing extend beyond the realm of local processing to include basic perceptual functions, such as detection, recognition and discrimination, as a result of enhancements in bottom-up, feed-forward perceptual operations. Although both accounts predict a focus on local perceptual features, WCC proposes that superiorities in local processing go hand-in-hand with difficulties in global processing, whereas EPF posits that autistic perception defaults to the local level, with global integration being intact but not mandatory (for a fuller discussion of EPF see Wang et al. 2007).

In response to these divergent predictions, a number of studies have directly tested the veracity of the theories by using drawing tasks. Sheppard et al. (2009b) studied drawing strategies to assess whether adolescents with autism organised their copied renditions according to an appreciation of the whole figure, or on a part-by-part basis. Autistic participants were no more likely to use a local strategy than those without autism and, since there was no group difference at the global level (also see Jolliffe and Baron-Cohen 2001; Ropar and Mitchell 2001), the authors argued that these data provided evidence for the EPF account. In contrast, other studies have reported clear difficulties in producing the global properties of drawings, which lie in favour of the WCC account. For example, in their copying task, Booth et al. (2003) found that autistic children were more likely to begin their renditions with a local detail, compared to both typical children and children diagnosed with Attention Deficit Hyperactivity Disorder. Autistic children were also more likely to draw in a piecemeal fashion, yielding a final global configuration that was incorrect. Similarly, Drake and Winner’s (2011) autistic savant artist (a 10-year-old boy of average intelligence) used a local strategy to complete drawings of three-dimensional objects to a greater extent than a comparison group of children with autism but without a drawing talent. He also performed particularly well on tasks of local perception (embedded figures, block design) and poorly on a global task (classification of impossible figures).

It is therefore clear that evidence from existing studies fails to yield consistent support for either account of visuospatial processing in autism. Furthermore, some studies report no group differences in drawing speed or accuracy (e.g., Eames and Cox 1994; Mottron et al. 1999) and others report *poorer* performance in autistic groups (e.g., Booth et al. 2003). There are a number of potential reasons for these differences. First, autism is a condition that involves substantial heterogeneity. As a result, neither the genetic (e.g. Ronald et al. 2006) nor the behavioural and cognitive profile (e.g. Pellicano 2010) is likely to be universal. Second, different studies have selected comparison groups according to different principles. So, for example, whereas Mottron et al. (1999) matched their groups according to chronological age and non-verbal mental age, Sheppard et al. (2009a, b) also matched theirs according to verbal mental age. The ability to accurately draw real objects has been related to vocabulary skills, especially in younger children (e.g., Toomela 2002), and so differences in the verbal skills of autistic participants might impact upon the behaviours measured. Third, and most importantly, the nature of the drawing tests themselves have likely given rise to these differences in performance. Some studies have used two-dimensional figures whereas others have used three-dimensional figures. The inclusion of dimensionality is a relatively advanced component of drawing, especially for children (Willats 1977), which may mask more subtle indices or variation. Equally, some studies have used meaningful stimuli whereas others have used more abstract ones. When meaning is not controlled for, it may introduce additional conceptual components that affect the planning and production of the drawing (van Sommers 1984). Furthermore, not all studies include blind rating of participant drawings. Preconceptions of group differences can affect the assessment of drawing output, irrespective of the measures used (Leek et al. 2000). Finally, studies have tended to employ dependent measures that conflate global and local processing, making it difficult to dissociate their impact. For example, beginning drawings at the local level (e.g., Booth et al. 2003; Drake and Winner 2011) could either reflect enhanced local processing or poor global processing.

We suggest that some of these problems can be overcome by adopting methods and approaches used to study similar behaviours in neuropsychological patients (i.e., individuals who have sustained neurological damage). Graphic production tasks are ubiquitous in patient assessment and there has been much interest in devising objective quantitative measures of drawing that can inform the delineation of fine-grained distinctions between both functional components of behaviour and diagnostic categories themselves (Leek et al. 2000; Smith 2009). In the present study, we therefore used a drawing task that was originally designed to assess the influence of grouping cues on drawing impairments in constructional apraxia, an acquired dysfunction of the ability to construct a coherent global form from local elements (in the absence of a perceptual or motor deficit). This is most likely to be observed in tasks such as drawing or arranging blocks and is associated with damage to parietal and frontal cortices (see Guérin et al. 1999). Smith and Gilchrist (2005a) presented two adult patients with figures constructed of four L-shaped corner elements, arranged to form a square. Grouping was manipulated by skewing two or more of the part elements, disrupting good continuation. Patient copies were scored according to their reproduction at both the local level (i.e., the presence or absence of local elements) and the scalar properties of the global form (i.e., internal angular accuracy, height-width ratio). Although the patients performed similarly on other qualitative measures of drawing production, measurements revealed an important dissociation between them: patient ECR tended to complete across gaps between elements to form a global square form, whereas patient RA represented individual part elements but with a poor global configuration (akin to some of the autistic copies illustrated by Booth et al. 2003). This study provided evidence for a finer-grained distinction within the broad category of constructional apraxia and also showed that local and global processing can be dissociated in the visuomotor domain.

In the present study, we used Smith and Gilchrist’s (2005a) methodology to assess drawing production in children on the autism spectrum. The benefits of using this task were that it: (1) provided a fine-grained parametric approach that is specifically focused on the impact of grouping on drawing production, enabling study of the process in the absence of potentially confounding factors such as meaning or linear perspective; (2) allowed us to dissociate global–local processing by yielding several variables of interest, tapping either local processing or global processing, but not both; and, (3) enabled blind and detailed objective assessment of the drawings. Copies were compared between a Grouped condition, where gestalt properties of good continuation favoured a strong whole, and a Skewed condition, where those cues were reduced by rotating some or all of the local elements. Critically, the vertices of the elements always formed the same square configuration, and we measured the deviation from this global square. If autistic children demonstrate a reduced appreciation of the gestalt properties of the image, as predicted by WCC, then we would expect the accuracy of their copies to be less affected by the reduction of grouping cues than that of typical children of similar age and ability. Insensitivity to grouping cues in children with autism would also be manifest in reduced scalar accuracy of the global configuration compared to typical children. If, however, autistic children exhibit global accuracy equal to that of typical children, and are similarly affected by the reduction of global cues, then this would provide evidence for the EPF account. Since both theories predict strength in the processing of local information, one should expect autistic children to perform more accurately than typical children in the production of part elements (i.e., fewer omissions of line segments).

In addition to the drawing task, we also investigated individual differences in drawing ability by examining the relationship between our key drawing indices (accuracy of global and local reproduction) and performance on measures tapping processes thought to underpin drawing ability (e.g., local processing and planning ability). Local processing was measured using the Children’s Embedded Figures Test (Witkin et al. 1971). Autistic children have repeatedly been shown to outperform typical children on this test (e.g. Pellicano et al. 2006; although see White and Saldaña 2011) and so it was of particular interest to assess whether any perceptual strength in the detection of local form would be reflected in visuomotor performance on our drawing task. Planning ability was measured using the Tower of London task (based on Shallice 1982). Harris and Leevers (2000) argue that poor planning abilities are responsible for some drawing difficulties in autism. However, Booth et al. (2003) found that planning abilities were unrelated to drawing performance in their tasks, despite the fact that such planning was poorer in their autistic sample. We therefore examined whether planning ability might be related to the more subtle metric indices of drawing that we were measuring.

## Method

### Participants

Participant descriptives are shown in Table 1. 21 children on the autism spectrum (two girls) and 21 typical children (three girls) aged between 8 and 14 years participated in this study. All children were recruited via local community contacts in the South West region of the United Kingdom. Autistic children had received an independent clinical diagnosis of either autism (n = 15) or Asperger syndrome (n = 6), according to DSM-IV-TR (APA, 2000) or ICD-10 (WHO 1992) criteria, and further scored above the threshold for autism spectrum disorder on the Social Communication Questionnaire (SCQ; Rutter et al. 2003) and on the Autism Diagnostic Observation Schedule—Generic (ADOS-G; Lord et al. 1999) (see Table 1).

**Table 1:** Descriptive characteristics for children with autism and typically developing children

Measures	Autistic children (n = 21)	Typically developing children (n = 21)	Group difference (*p* value)
Gender (n males: n females)	19:2	18:3	
Age (years; months)			
Mean (SD)	10; 7 (1; 8)	10; 11 (2; 0)	.59
Range	8; 6–14; 4	8; 2–14; 8	
Verbal ability^a^			
Mean (SD)	102.33 (16.69)	106.10 (14.37)	.44
Range	80–137	82–132	
Nonverbal ability^b^			
Mean (SD)	36.14 (7.95)	36.24 (4.84)	.96
Range	25–48	24–45	
SCQ score			
Mean (SD)	25.43 (5.63)	4.76 (3.03)	<.001
Range	18–35	1–11	
ADOS-G total score^d^			
Mean (SD)	10.64 (3.43)		
Range	7–20		

^a^Standard scores on the British Picture Vocabulary Scale—2nd Edition (BPVS; ref)

^b^Raw scores on Raven’s Standard Progressive Matrices (Raven et al. 1991)

^c^Total scores on the Social Communication Questionnaire (SCQ; Rutter et al. 2003)

^d^ADOS-G = Autism Diagnostic Observation Schedule—Generic (Lord et al. 1999); Elevated scores on the SCQ and ADOS-G reflect greater levels of autistic symptomatology

Children were included in this study only if they achieved a standard score of 80 or above on the British Picture Vocabulary Scale—2nd Edition (BPVS; Dunn et al. 1997) and were free of medication. Typical children neither scored above the cut-off for autism on the SCQ (indicative of low levels of autistic symptomatology) nor had a current or past developmental condition, as reported by parents. The autism and typical groups did not differ significantly in terms of chronological age, *t*(40) = 0.54, *p* = .590, verbal ability as measured by the BPVS, *t*(40) = 0.78, *p* = .440, or nonverbal ability, *t*(40) = 0.05, *p* = .960, as measured by the Raven’s Standard Progressive Matrices (Raven et al. 1991).

### Procedure

#### ‘Drawing Corners’ Task (Based on Smith and Gilchrist 2005a)

**Fig. 1:**
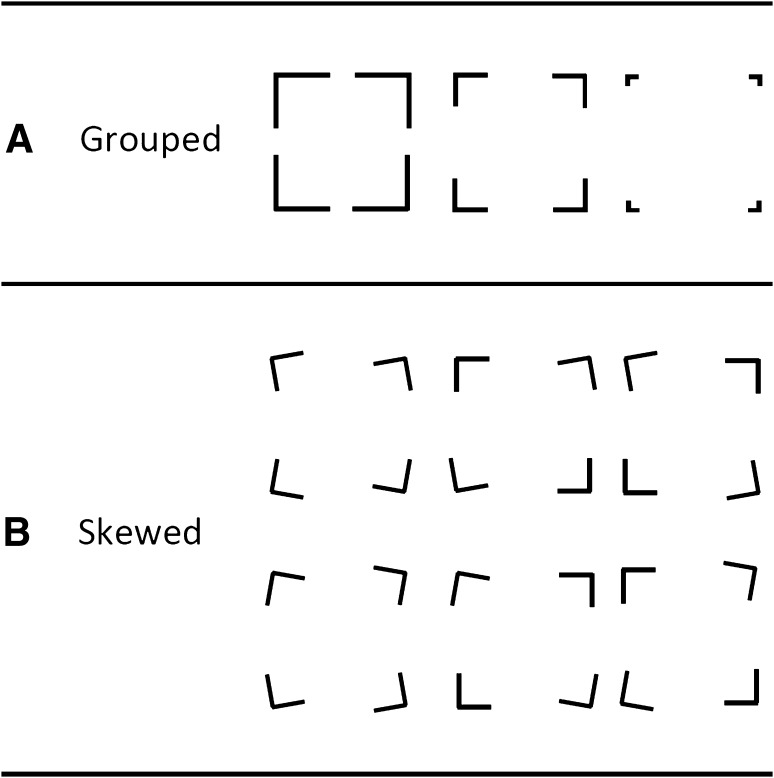
Stimuli for the drawing corners task. Each of the Grouped stimuli (**a**) were presented four times, and each of the Skewed stimuli (**b**) were presented twice

Two different configurations of stimuli were created with the printed figure in the upper portion of an A5 sheet of paper (portrait orientation). The first, Grouped set consisted of four corners arranged to form a 6 × 6 cm square (see Fig. 1a). To provide some variability (and therefore to reduce the monotony of the task for children), there were three different sizes of gaps between the corners: 1, 3 and 5. Each gap size was presented four times, yielding 12 trials for the Grouped condition.

The second, Skewed set of stimuli consisted of corners whose orientation had been systematically rotated by 10° (see Fig. 1b). Rather than the adjustment of gap size, task variability in this condition was achieved by specifying six different types of stimulus, depending on the number of elements that were skewed (two or four), the direction of the skew (clockwise or anticlockwise) and the location of the skewed elements (top-left and bottom-right, bottom-left and top-right, or all elements). Half of the trials within each orientation contained only two rotated corners; the remaining trials had all four rotated. Despite rotations, the vertices of all of the elements formed the same 6 × 6 cm square. Each of these stimuli were presented twice, and all 24 trials were presented in a different randomised order for each child.

Children were told, “I have some pictures that I would like you to copy.” The first stimulus was placed in front of the participant. They were instructed to “copy the picture as accurately as possible in the space below”. When the participant had finished copying, they were given the next stimulus. This procedure continued until all 24 trials had been completed. There was no time limit for this task, and drawing time was not recorded, although children took approximately 10 min to complete all 24 trials.

**Fig. 2:**
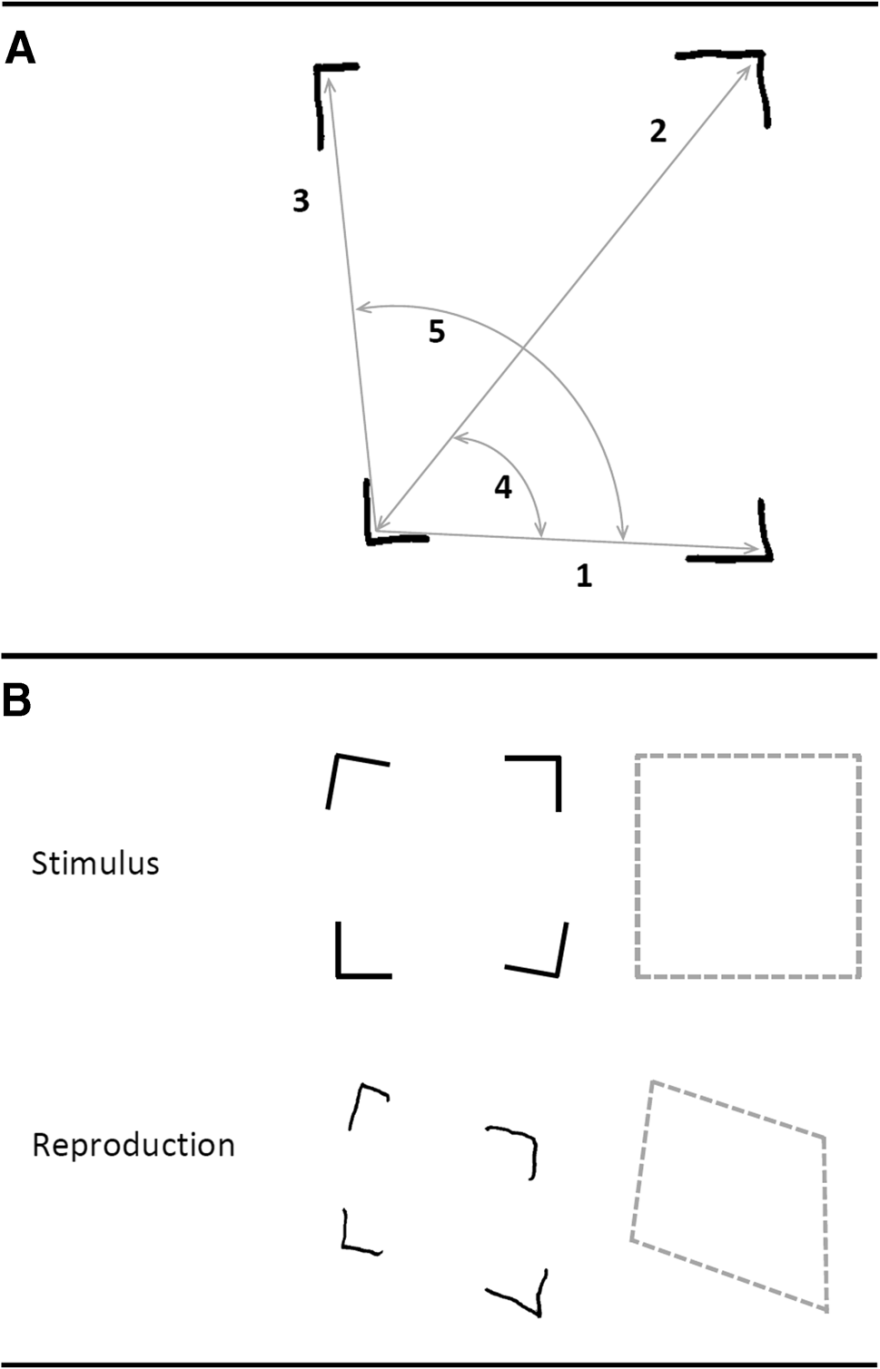
Details of global accuracy measures. **a** Measurements that were taken by coders: *1* Distance between bottom-left and bottom-right vertices; *2* Distance between bottom left and top right vertices; *3* Distance between bottom-left and top-left vertices; *4* Angle between imaginary lines *1* and *2*; *5* Angle between imaginary lines *1* and *3* **b** Illustration of the global square formed by the vertices of the stimulus, compared with global configuration formed by a participant’s reproduction of the model. Global measures compare internal angular accuracy and height/width ratio of the copy’s configuration to that of the model. Note that the precise orientation of the configuration is not included in the analyses

All drawings were independently coded by one of two trained raters who were blind to participant details (i.e., group, age, gender); 5 % of these codes were second coded by a third trained rater. Using the same technique as Smith and Gilchrist (2005a), measures of the global qualities of drawings were derived from angle and length measurements taken directly from the drawing (as illustrated in Fig. 2a). These measurements were then processed trigonometrically to derive the relative locations of each vertex. Since, in both the Grouped and Skewed conditions, the vertices of the model always formed a 6 × 6 cm square, global error was conceived as the deviation away from that square configuration, irrespective of either the local orientation of individual corner elements or the angle between the two lines that formed each corner element.

By comparing the scalar properties of the square formed by the vertices of the copy to those in the model (i.e., deviations from a 6 × 6 cm square), we formulated two global metrics that could be applied to both Grouped and Skewed stimuli. These were (1) the *internal angular accuracy* of the square (expressed as an error in degrees) and (2) its *height/width ratio*. For this latter measurement, a score of 1 would indicate a perfect square, with equal height and width (i.e., a ratio of 1:1), whereas a deviation from a square would be indicative of a more oblong shape (i.e., a positive value if the shape is taller than it is wide, and a negative value if the shape is wider than it is tall). This principle is illustrated in Fig. 2b.

Local scores were more detailed than those calculated by Smith and Gilchrist (2005a) and were derived from a checklist that required the raters to code properties of each local corner element (e.g., number of lines, orientation) as well as whether there were omissions of corner elements or the individual line segments thereof. The qualities of local elements were systematically scored according to three key factors: (i) whether local corners were formed of two line segments; (ii) whether corners formed an angle between 67.5°–12.5° (i.e. within 45° of the correct 90° angle); and (iii) whether the corners were correctly oriented (i.e. a ‘top-left’ vertex should have one line extending downwards and another rightwards). Each corner was scored regarding the presence (score of ‘1’) or absence (score of ‘0’) of each of these factors, yielding a maximum score of three for each factor and a maximum total score of 12 (summing across all four corners).

#### Children’s Embedded Figures Test (CEFT; Witkin et al. 1971)

The CEFT was used to measure local–global information processing. The test includes two sets, incorporating three practice trials and 25 test trials. In each set, children initially were shown a cardboard cut-out of a target shape (set A: triangle; set B: house) and asked to find this shape hidden in a larger meaningful figure (e.g., a pram) as quickly as possible (set A: 11 trials; set B: 14 trials). Response latencies and accuracy were recorded for each trial. Children were given a maximum of 30 s to locate the target stimulus on each trial. One point was given for each trial on which they successfully located the hidden target. If the triangle was not located within the time limit, then an error was recorded, and the maximum time (30 s) was given for that trial. The dependent variable of interest was time taken to find the hidden figure. Autistic children typically perform faster on this task than typical children do, purportedly because they are not captured by the global image, allowing them to focus on the individual elements and quickly find the hidden target (Shah and Frith 1983). As such, fast times on the CEFT reflect good local processing (and, hence, poorer global processing).

#### Tower of London Task

In this test of higher-order planning ability (based on Shallice 1982), children were presented with a wooden pegboard consisting of three vertical pegs of increasing size (small, medium, big) and given three coloured beads (red, white, black), which they arranged in a particular configuration (start state). They were then shown a picture of the beads in a different configuration (goal state). Children were instructed to move the beads from the start state to the goal state in as few moves as possible (shown clearly on the bottom of each picture). They were also told that they (a) could only move one bead at a time and (b) must not place beads on the table. There were three practice trials followed by problem sets of increasing difficulty, including four trials of one-, two-, three-, four-move, and five-move problems. Testing ceased if a child failed all of the problems within a set. Successful performance on this task required children to identify the sequence of steps required to solve a novel problem. Children were therefore given one point for each trial if they reached the goal state within the minimum number of moves and without violating any rules (maximum score = 20). Higher scores reflect better planning ability.

#### General Procedure

Ethical approval for this study was granted by the University of Bristol’s Faculty of Science Human Research Ethics Committee. Parents of all children gave informed written consent for their child’s participation; children also gave their assent to take part. Children were seen individually on a single occasion at the university. Tests of verbal and nonverbal ability were always administered first followed by the *drawing corners* game and other behavioural measures, the order of which was randomized across participants.

## Results

Examples of children’s copies are illustrated in Fig. 2. These were measured to produce global accuracy indices of internal angular error and height/width error (of the square formed by the four vertices). Owing to the sensitivity of these measurements, inter-rater reliability was assessed: The intraclass correlation for double-coded measurements indicated excellent inter-rater reliability, *r*_ic_(255) = 0.99, *p* < .001. Local accuracy measures were categorically coded on the basis of the presence or absence of features, their relationship with each other, and their orientation on the page. Analyses were performed using SPSS version 22 software (IBM International).

### Global Properties

**Fig. 3:**
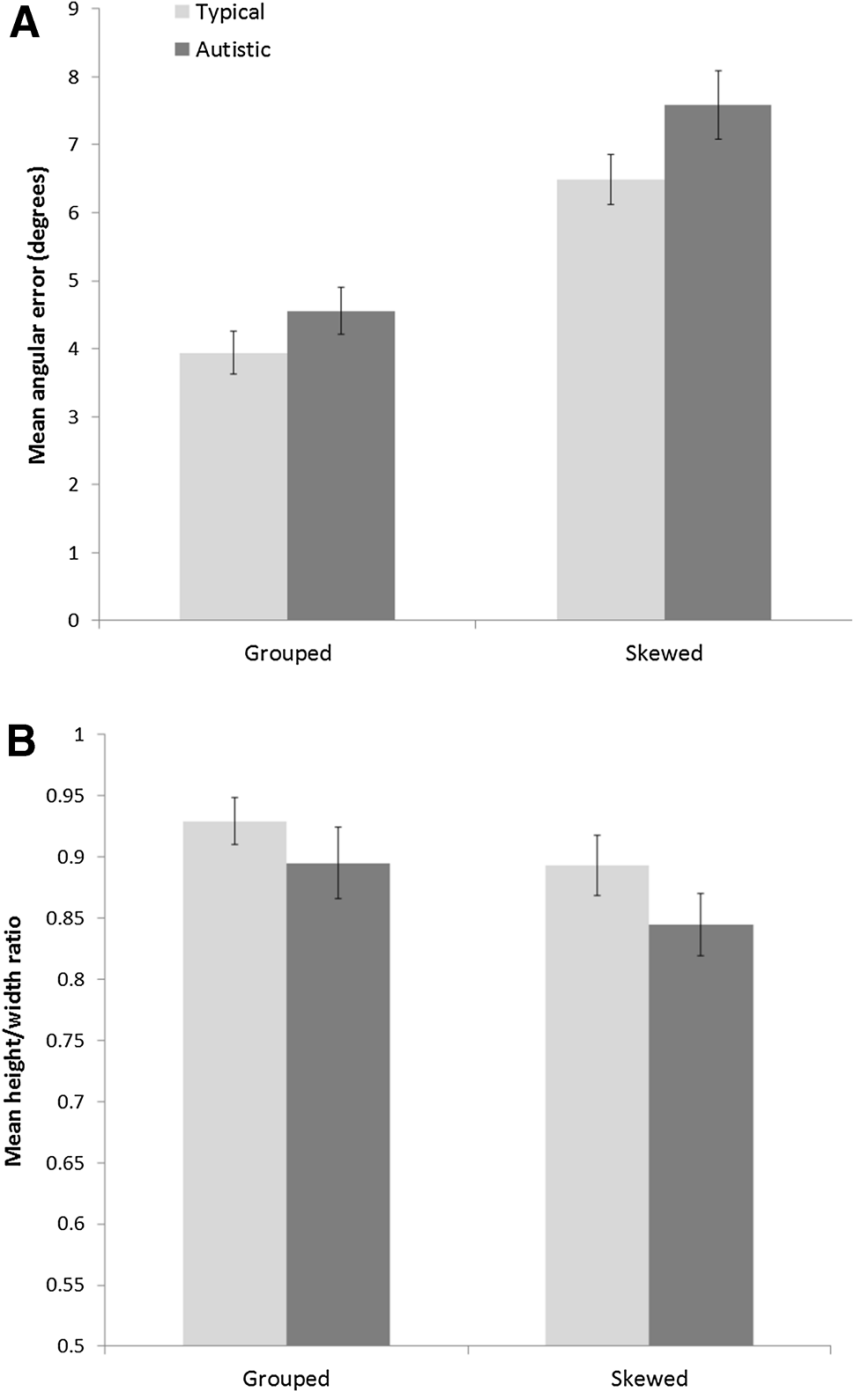
Graphs to show global accuracy of children’s drawings: average angular error (**a**) and average height/width ratio (**b**). *Error bars* represent standard error of the mean

Global data are illustrated in Fig. 3. Mean angular error (in degrees) was initially subjected to a 2 (Configuration: Grouped, Skewed) × 2 (Group: autistic, typical) mixed-design ANCOVA, with age (in months) included as a covariate. There was a significant effect of configuration, with greater angular error for Skewed compared to Grouped stimuli, *F*(1, 40) = 6.23, *p* < .020, η_*p*_^2^ = .140. There was no significant main effect of group, *F*(1, 40) = 2.69, *p* = .110, η_*p*_^2^ = .060, and no configuration × group interaction, *F* < 1. Angular error was unaffected by age of participants, *F* < 1, and there was no age × configuration interaction, *F* < 1.

We performed the same ANCOVA on our second global quality measure, mean height/width ratio. This analysis revealed no main effects of configuration or group and no configuration × group interaction, all *F*s < 1. Height/width ratio was unaffected by age, *F* < 1, and there was no age × configuration interaction, *F* < 1.

### Local Properties

Every drawing received a score out of 12, with each of the four corners being scored on whether they were (1) constructed of two elements, (2) appropriately angled, and (3) appropriately oriented). The total score was expressed as a percentage, it is clear that the majority of copies were accurate at the local level (see Table 2). Cautious of the high number of completely accurate responses (i.e. 100 % across factors and corners), data for Grouped and Skewed conditions were submitted to Shapiro-Wilks analysis and neither was found to be normally distributed (Grouped: W = .790, *p* < .001; Skewed: W = .940, *p* < .050). In the absence of a non-parametric alternative for a mixed-design ANOVA, we subjected the data to a series of focused non-parametric analyses. To test for group differences in each condition we conducted Mann–Whitney analyses, which revealed no difference between autistic and typical children for Grouped stimuli (*U* = 161, *p* = .130) or for Skewed stimuli (*U* = 167, *p* = .180). To test for within-group effects of condition, related-samples Wilcoxon tests were conducted, separately for each group, to confirm whether there was a difference between Grouped and Skewed stimuli. In this case, autistic children showed a significant difference between conditions, with higher local scores for Grouped stimuli (*Mdn* = 97.9, *IQR* = 13.2) than for Skewed stimuli (*Mdn* = 91.7, *IQR* = 20.1): *Z* = −3.14, *p* = .002. This was the same for typical children, who demonstrated higher local scores for Grouped stimuli (*Mdn* = 98.6, *IQR* = 7.64) compared to Skewed stimuli (*Mdn* = 93.8, *IQR* = 13.2): *Z* = −3.23, *p* = .001. Note, however, that these data may be subject to a ceiling effect and so all analyses should be interpreted with some caution.

**Table 2:** Median local scores for each category, expressed as a percentage of the total achievable score (interquartile range in parentheses)

Local measure	Condition	Autistic children	Typically developing children
Corners formed from two connected segments	Grouped	100 % (0)	100 % (0)
	Skewed	100 % (0)	100 % (0)
Corners form an angle of 67.5°–112.5°	Grouped	100 % (0)	100 % (0)
	Skewed	93.8 % (12.5)	100 % (6.3)
Corners correctly oriented	Grouped	100 % (6.3)	100 % (6.3)
	Skewed	87.5 % (18.8)	93.8 % (12.5)

### Individual Differences

We took measures of children’s abilities that might be expected to underpin drawing, namely local–global processing (measured by the CEFT) and planning (measured by the Tower of London task). As expected, autistic children showed significantly faster search times on the CEFT (*M* = 5.45; *SD* = 3.69) than typical children (*M* = 9.23; *SD* = 4.14), *t*(40) = 3.13, *p* = .003. There was, however, no significant differences in the scores on the Tower of London task (number of correct trials) between autistic (*M* = 12.95; *SD* = 1.91) and typical children (*M* = 13.24; *SD* = 3.15), *t*(40) = 0.36, *p* = .720.

We assessed whether these skills were related to global accuracies using regression analyses for each group separately, given that we have a priori reasons to expect different patterns of relationships in each group. Our first index of global processing, mean angular error (across both conditions), was entered as the dependent variable in a hierarchical regression. In the autistic group, we initially entered children’s age, verbal ability (BPVS raw score) and non-verbal ability (Raven’s raw score). These variables failed to make a significant contribution to the model, Δ*R* ^2^ = .150, Δ*F* (3, 20) = 1.00, *p* = .420. Next, we entered children’s CEFT performance (mean search time) in the second step of the analysis, which made a significant contribution to the model. Independent of age, verbal ability and non-verbal ability, autistic children who performed faster on the CEFT (indicative of better local processing) produced copies with greater angular error in the global configuration (*β* = −.640, *p* = .002, Δ*R* ^2^ = .400, Δ*F*(1, 20) = 14.02, *p* = .002). Finally, we entered children’s Tower of London scores (number of correct trials) into the third step of the model, but this made no additional contribution to the model, Δ*R* ^2^ = .006, Δ*F*(1, 20) < 1.

In the typical group, the same regression analysis revealed no significant contributions of age, verbal ability and non-verbal ability, Δ*R* ^2^ = .240, Δ*F*(3, 20) = 1.77, *p* = .190. Entering CEFT performance in the second step of the analysis made a significant contribution to the model, but in the opposite direction to that of the autistic children: those TD children who performed more slowly on the CEFT (indicative of poorer local processing) produced copies with greater angular error (*β* = .567, *p* = .009, Δ*R* ^2^ = .270, Δ*F*(1, 20) = 8.76, *p* = .009). Tower of London scores made no additional contribution to the model, Δ*R* ^2^ = .002, Δ*F*(1, 20) < 1. The respective predictive power of CEFT and Tower of London scores remained the same if they were entered into the models in the reverse order.

Similar hierarchical regression analyses were conducted on children’s height/width ratio data, as an alternative index of global processing: there were no significant contributions of either variable for autistic (CEFT: *β* = −.010, *p* = .130; Tower of London: *β* = .010, *p* = .690) or typical children (CEFT: *β* = −.010, *p* = .120; Tower of London: *β* = .010, *p* = .130).

We also examined the relationship between individual differences in children’s local scores (across conditions) and their performance on the CEFT and Tower of London by performing non-parametric correlational analyses. The relationship between children’s CEFT performance and local scores was not significant for autistic, *r*_s_ = .410, *p* = .060 or typical children, *r*_s_ = .410, *p* = .060. Tower of London scores were also unrelated to local scores for both autistic (*r*_s_ = .190, *p* = .400) and typical (*r*_s_ = .330, *p* = .140) groups.

Finally, to assess whether there was a consistent relationship between global and local performance within each group, we performed non-parametric correlational analyses on angular error (as the most illustrative global measure) and local accuracy (across conditions). A significant positive relationship between global error and local accuracy (i.e., such that increasing global error would be associated with increasing local accuracy) would be indicative of a trade-off between local and global performance. Although we found a significant relationship between these variables, it was in the opposite direction than expected for both autistic children, *r*_*s*_ = −.560, *p* = .009, and typical children, *r*_*s*_ = −.480, *p* = .030, with greater local accuracy being associated with less global error.

## Discussion

We assessed whether autistic children’s drawing production was affected by the global qualities of the to-be-copied model. Children on the autism spectrum were just as likely as typical children to be affected by manipulations of gestalt properties. When some or all of the corner elements were skewed, thus reducing the good continuation of the global square, both autistic and typical children produced copies that had greater error in the scalar properties of the global configuration (i.e., greater internal angular error and greater deviation of height/width ratio from 1:1). Across all analyses, there were no significant effects of group or any group × condition interactions. Furthermore, neither group demonstrated a trade-off between global and local performance—local accuracy was, in fact, related to less global error for autistic and typical children alike.

Overall, this pattern of results suggests that autistic children are sensitive to manipulations of global structure. When grouping cues were manipulated, the global qualities of their copies were reliably affected, and to a similar extent as their typically developing counterparts. These findings are in accordance with previous research that has reported no group difference in drawing performance at the global level (e.g., Mottron et al. 1999; Sheppard et al. 2007). Taken together, these findings speak against the WCC account (Happé and Frith 2006), which states that individuals on the autism spectrum are less sensitive to global structure than typical individuals. Furthermore, our findings do not support those of Booth et al. (2003), who reported poor configural properties of reproductions. One reason for the discrepancy in findings is that our stimuli were representationally simpler (i.e., two-dimensional geometric forms) and were not supported by a semantic interpretation. For example, whilst Booth et al. (2003) asked participants to draw, say, a house, we simply asked children to copy what they could see. Despite the relative simplicity of our stimuli, however, the global manipulations were sufficiently strong to modulate copying behaviour in both typical and autistic children, which warrants confidence in our findings. It is perhaps the case that reported drawing differences between autistic and typical children are a result of semantic factors relating to the planning of drawings (see van Sommers 1984), rather than purely visuospatial components.

There is, however, an aspect of our results that could be taken as support for the WCC account, namely, autistic children’s CEFT performance. First, they were significantly faster to find the hidden figure than typical children, replicating previous findings (Jolliffe and Baron-Cohen 1997; Pellicano et al. 2006) and demonstrating a local bias in the visual domain (i.e. search) that was not observed in the visuomotor domain (i.e. drawing accuracy). Second, there was a significant relationship between CEFT performance and copying accuracy in the autistic children in the predicted direction: those who displayed a local bias in the task, by locating the local shape more rapidly, were more likely to demonstrate global inaccuracies in their copies. Interestingly, this pattern was reversed in typical children, such that those who displayed a local bias in the task were *less* likely to demonstrate global inaccuracies. This CEFT-drawing relationship suggests that relative strengths in one visuospatial task (emphasising the local level) might be associated with relative weaknesses in another (requiring attention to the global level), in a manner consistent with the predictions of WCC. This was observed for our autistic sample, and the relationship is in accordance with previous findings that local bias in the CEFT is associated with inefficiencies in large-scale spatial learning (Pellicano et al. 2011). It is therefore possible that the autistic children were using different cognitive strategies to produce their copies. Although this may have only been evident in a discrete aspect of their global performance, the unique negative relationship between global accuracy and local bias may be indicative of a different approach to the analysis and reproduction of figures. The positive relationship between CEFT performance and average angular error in typical children is less in-line with our predictions, although it may instead reflect a general relationship between performance levels across tasks (i.e. those children who performed more efficiently in the CEFT were also more accurate at the copying task) (see Pellicano 2010, for a discussion of the relationship between CEFT and other skills). In future research, it would be beneficial to record the time-course of children’s copying behaviour, which might shed further light on potential qualitative differences between autistic and typical children’s drawing skills.

In contrast with CEFT performance, planning abilities (as measured by children’s Tower of London scores) did not predict performance in either group. Furthermore, unlike Booth et al. (2003), we did not find a group difference on planning abilities, although this could be related to the fact that we used a different measure. While they measured planning in relation to the drawing task itself, our measure of higher-order planning (the Tower of London) required children to plan a longer sequence of moves. Our assay of planning was therefore potentially more difficult—for both groups of children—than the one used by Booth et al. (2003).

The Enhanced Perceptual Functioning (EPF) account of visuospatial behaviour in autism (Mottron et al. 2006) explicitly states that superiorities at the local level are distinct from global processing skills, which are held to be intact. The findings regarding the relationship between autistic children’s drawing performance and the CEFT are therefore contrary to those predictions. Nevertheless, the overarching picture created by the data is one of no group differences in drawing performance—neither in terms of their attention to the global configuration nor to the individual constituent elements. These data, therefore, do not support an argument for local superiority in drawing production, as proposed by previous graphic production studies that favour the EPF account (e.g. Mottron et al. 1999; Sheppard et al. 2009b). However, it is important to note that these data may be subject to a ceiling effect, since the majority of drawings were produced without error at the local level. Future studies should, therefore, take an even more fine-grained approach, particularly with regard to angular deviation at the local level, to examine further potential differences in drawing skills. More subtle differences between groups may also be observable at a graphomotor level of explanation. For example, greater error in the Skewed condition not only relates to the reduction of grouping cues but also to the presence of oblique lines, which were not present in the Grouped condition. Oblique lines are more difficult to plan or execute than horizontal and vertical lines (Broderick and Laszlo 1987, 1988), although it is interesting that there did not seem to be any observable differences between autistic and typical groups in the representation of oblique orientations (c.f. Smith and Gilchrist 2005b)

Taken together, these findings in the visuomotor domain suggest that the two main accounts of visuospatial processing in autism (WCC and EPF) may not sufficiently capture drawing behaviour. It might therefore be useful for developmental psychologists to reconceptualise their approach to hierarchical perception and incorporate existing models of attention and perception from allied fields of psychology. For example, Humphreys (1998) draws the distinction between within-object coding (i.e., parallel coding of parts within objects) and between-object coding (i.e., parallel coding of multiple separate objects). These processes are conceptually similar to, respectively, global and local processing. However, in developmental accounts, global processing tends to be conceived as a parallel process, whereas local encoding is thought to rely on a serial individuation of separate smaller elements. The account put forward by Humphreys (1998) states that both processes occur in parallel (with global perception not being reliant upon the local level) and can be separately disrupted by damage to different brain regions. A recent meta-analysis of hierarchical visual processing in autism echoes this viewpoint (Van der Hallen et al. 2015). The authors report neither superior local visual processing nor a deficit in global processing in autism, and instead report a difference in the speed at which autistic people perceive the global form when there is interference from local details (e.g., in the Navon task and the CEFT). Our findings suggest that this distinction extends to the visuomotor domain, and future work in this area should aim to clarify whether perceptual differences between groups are reflected in the motor components of drawing production.

It may therefore be beneficial to take a wider perspective when constructing theories of visuospatial behaviour in autism, incorporating findings and theories from similar explorations in different populations. A recent study by D’Souza et al. (2016) compared global and local performance in participants with autism, Williams Syndrome and Down Syndrome, across a battery of tasks. They found both local and global biases in all three populations, depending on the nature of the task, and argued that a broad global/local distinction between syndromes is inappropriate. This suggests that it may be useful to consider the encoding of spatial relations within and between objects as something that is separable from the perception and detection of local features (see also Farran et al. 2003). By considering how these processes interact with each other, we can therefore move towards an account of visuospatial behaviour that encapsulates the variety of reported findings. It should also ensure that autism research is appropriately informed by advances in vision research, neuropsychology, and cognitive neuroscience, just as those areas have been fundamentally informed by autism research.
